# Quality of asthma care under different primary care models in Canada: a population-based study

**DOI:** 10.1186/s12875-015-0232-y

**Published:** 2015-02-14

**Authors:** Teresa To, Jun Guan, Jingqin Zhu, M Diane Lougheed, Alan Kaplan, Itamar Tamari, Matthew B Stanbrook, Jacqueline Simatovic, Laura Feldman, Andrea S Gershon

**Affiliations:** Child Health Evaluative Sciences, The Hospital for Sick Children, 555 University Avenue, Toronto, Canada; Institute for Clinical Evaluative Sciences, Toronto, Canada; Dalla Lana School of Public Health, University of Toronto, Toronto, Canada; Institute of Health Policy, Management and Evaluation, University of Toronto, Toronto, Canada; Department of Medicine, Queen’s University, Kingston, Canada; ICES – Queen’s, Kingston, Canada; Family Physician Airways Group of Canada, Warwick, Canada; Stonegate Community Health Centre, Toronto, Canada; Toronto Western Research Institute, University Health Network, Toronto, Canada; Sunnybrook Health Sciences Centre, Toronto, Canada

**Keywords:** Asthma, Quality of care, Performance measures, Health indicators, Health services use

## Abstract

**Background:**

Previous research has shown variations in quality of care and patient outcomes under different primary care models. The objective of this study was to use previously validated, evidence-based performance indicators to measure quality of asthma care over time and to compare quality of care between different primary care models.

**Methods:**

Data were obtained for years 2006 to 2010 from the Ontario Asthma Surveillance Information System, which uses health administrative databases to track individuals with asthma living in the province of Ontario, Canada. Individuals with asthma (n=1,813,922) were divided into groups based on the practice model of their primary care provider (i.e., fee-for-service, blended fee-for-service, blended capitation). Quality of asthma care was measured using six validated, evidence-based asthma care performance indicators.

**Results:**

All of the asthma performance indicators improved over time within each of the primary care models. Compared to the traditional fee-for-service model, the blended fee-for-service and blended capitation models had higher use of spirometry for asthma diagnosis and monitoring, higher rates of inhaled corticosteroid prescription, and lower outpatient claims. Emergency department visits were lowest in the blended fee-for-service group.

**Conclusions:**

Quality of asthma care improved over time within each of the primary care models. However, the amount by which they improved differed between the models. The newer primary care models (i.e., blended fee-for-service, blended capitation) appear to provide better quality of asthma care compared to the traditional fee-for-service model.

**Electronic supplementary material:**

The online version of this article (doi:10.1186/s12875-015-0232-y) contains supplementary material, which is available to authorized users.

## Background

Common worldwide challenges to the delivery of primary care have included limited time spent with patients, minimal funding and physician shortages. Primary care systems have attempted to alleviate the burden of some of these issues, and as a result different primary models have been implemented around the world, leading to an increased number of group-based practices and changes to remuneration. Recent primary care changes in Ontario, Canada have moved away from the traditional fee-for-service model, towards models that emphasize patient enrolment and provision of packaged services, such as blended fee-for-service, blended capitation and salaried models. These models are thought to provide a more comprehensive approach to patient care. Since this transition, much research has focused on structural aspects of the new models, such as patient enrolment or number of services provided. More recently, there has been an interest in the quality of care provided by these models. Russell et al. examined performance indicators specific to diabetes, congestive heart failure and coronary artery disease and found that physicians under salaried models provided superior quality of care to patients with those conditions [1]. Liddy et al. also found quality of diabetes treatment to be better in salaried models, whereas quality of preventative measures, such as smoking cessation, was better among blended capitation models [2].

Recently in Ontario, there has been a focus on improving the quality of asthma care. For example, the Primary Care Asthma Program has been implemented in over one-hundred primary care locations with the aim of providing tools to guide practitioners and patients in more effectively managing asthma [3-5]. However, despite these recent endeavours to improve the future quality of asthma care, little is known about the current quality of asthma care in Ontario, particularly among different primary care models. As Ontario is still in the process of a primary care change, a better understanding of quality of care among primary care models for particular conditions typically cared for at the primary care level, such as asthma, are of interest. The objective of this study was to use previously validated, evidence-based performance indicators to measure quality of asthma care over time and to compare quality of care between different primary care models.

## Methods

### Study population

The Ontario Asthma Surveillance Information System (http://www.sickkids.ca/Research/OASIS) is a population-based, longitudinal surveillance system that identifies and tracks individuals living with asthma in Ontario, Canada using four health administrative databases housed at the Institute for Clinical Evaluative Sciences (details in Additional file 1). Linkage of datasets using an encrypted version of the unique health insurance number given to all Ontario residents allows for examination of individuals’ complete health services use across health care domains. Health services claims identified from the databases from fiscal year 2006 (April 1, 2006 - March 31, 2007) to fiscal year 2010 (April 1, 2010 - March 31, 2011) were included. A previously validated case definition of asthma was used to identify individuals with asthma, and included those who have had at least two primary care visit claims for asthma in two consecutive years and/or at least one hospitalization for asthma [6,7]. This definition has been shown to have 89% sensitivity and 72% specificity in children (under 18 years old) and 83.8% sensitivity and 76.5% specificity in adults [6,7].

### Outcome measures – asthma performance indicators

Quality of primary care was measured using six validated, evidence-based asthma performance indicators from the linked databases described above [8]. Outcome measures included four process-related indicators: spirometry for diagnosis, spirometry for monitoring, reliever and controller medication prescription; and two outcome-related indicators: primary care visits and emergency department visits for asthma.

Spirometry for diagnosis was calculated as the percent of cases in which testing was performed in those ≥7years of age within 3.5 years of the presumed asthma diagnosis date (1 year prior and 2.5 years post asthma diagnosis) [9]. Spirometry for monitoring was calculated as the percent of cases in which testing was performed in those ≥7 years of age in the past 12 months. It has been suggested that pulmonary function measures should be followed over the patient’s lifetime to detect a decline of lung function and to track the rate of decline longitudinally.

Medication reliever and controller prescription indicators were calculated as the percent of individuals with asthma who were prescribed, respectively, short-acting β2-agonists (SABA) and inhaled corticosteroids (ICS) in the previous 12 months. It should be noted that while ICS remains the first-line controller therapy for all ages as recommended in most asthma management guidelines, physicians may adjust the ICS dose or discontinue ICS depending on levels of asthma control. Canadian Guidelines suggests that very mild, intermittent asthma may be treated with SABA as needed [10]. Medication data was only available for persons >65 years of age (12.1% of the asthma population in this study) and others eligible for the Ontario Drug Benefits Program, which includes those living in long-term care homes, those enrolled in home care programs, those with high drug costs relative to their income, and those receiving social assistance. Thus, findings of medication use should be interpreted with caution.

While many physicians diagnose individuals as having asthma based on the symptoms they present, such as wheezing, shortness of breath or cough, asthma guidelines recommend that asthma be diagnosed using spirometry. Once diagnosed, asthma is to be controlled with daily (versus intermittent) ICS use, thus we considered higher rates of spirometry and ICU prescription to reflect higher quality of care [11]. In contrast, lower SABA prescription rates were generally considered to reflect better asthma control and consequently higher quality of care. The number of canisters filled was calculated by total amount paid divided by unit cost of the respective medication. Less than or equal to 4 canisters per patient per year was thought to reflect better asthma control and ≥13 canisters per patient per year was thought to reflect poorer asthma control [12,13]. It should be noted that low use of SABA may also reflect suboptimal medical care or patient adherence in some cases, thus SABA findings should be interpreted with caution.

Primary care visits for asthma and emergency department visits for asthma were calculated per 100 patients with asthma in the past 12 months.

International Classification of Diseases (ICD) codes were used in the creation of the study population and to identify asthma health services use; specifically, ICD-9 (493) and ICD-10 (J45, J46) codes were used.

### Predictor variables – primary care practice models

The Ontario Ministry of Health and Long-Term Care (MOHLTC) has set criteria for each type of primary care practice model used by family medicine physicians in the delivery of care [2]. Physicians are remunerated either by fee-for-service or by one of the patient enrolment models: blended fee-for-service, blended capitation, or salary. Fee-for-service billing involves payment for every item or unit of care that physicians provide for their patients; physicians remunerated by fee-for-service may practice independently (solo) or be affiliated with a group (non-solo). The blended fee-for-service model remunerates physicians primarily through fee-for-service, but also includes incentives and bonuses for services to enrolled patients. The blended capitation model remunerates physicians based on number of enrolled patients, and on providing a basket of services to enrolled patients based on age and sex. Each additional service would be paid for by fee-for-service. Physicians working under the salaried model are remunerated based on the number of physicians within a group that provide services to a specific community. The definitions of practice models used came from the MOHLTC, and have been used previously by others [14]. Specific details can be found on the Health Force Ontario website: www.healthforceontario.ca/en/Home/Physicians/Training_|_Practising_in_Ontario/Physician_Roles/Family_Practice_Models/Family_Practice_Compensation_Models. These categories are mutually exclusive; physicians can only be listed in one model at any given time. All models were in effect for the entirety of the study period.

Physician demographic data came from the Institute for Clinical Evaluative Sciences (ICES) Physician Database. Fee-for-service practitioners were identified using Ontario Health Insurance Plan data and the ICES Physician Database, whereas physicians in the blended fee-for-service and blended capitation models were identified using Client Agency Program Enrolment data tables. Less than 1% of the study population failed to be linked to a practice model. Lack of access to community health centre data (one group within the salaried model) prevented the inclusion of the 73 community health centres in Ontario, which serviced approximately 4% of the population. Due to small sample sizes and a unique, potentially biasing geographic location, the Rural Northern Physician Group (the other group within the salaried model) was not included in this study. As community health centres and Rural Northern Physicians Groups are the only groups in Ontario under the salaried physician model, this study does not include data on the salaried model.

### Covariates and potential confounders

Potential confounding factors were adjusted for in a multivariable analysis [15]. Patient factors included sex, age group, socioeconomic status (inferred from neighbourhood income, as derived from postal codes and census data) and rurality (determined using postal code and based on living in a municipality with fewer than 10,000 people). Since some patients with asthma may also have chronic obstructive pulmonary disease (COPD) that may influence disease management [16], co-diagnosis of COPD was included as a covariate in the multivariable analysis. Individuals were defined as having COPD if they were ≥35 years of age and had at least one COPD hospitalization and/or one COPD ambulatory care claim (85% sensitivity & 78% specificity) [17]. Physician factors included sex, age, years practicing medicine and whether or not training occurred in Canada. Volume of registered asthma patients was also considered.

### Statistical analysis

A retrospective population-based cross-sectional study design was used. Six asthma indicator trends over time were described overall and stratified by primary care practice models. The equivalence test [18] was used to evaluate differences between groups for each of the patient and physician characteristics. All variables listed in Table 1 were included in the multivariable regression analysis. Two-level Poisson regression models with GEE (Generalized Estimating Equations) were used to account for clustering of patients within primary care models as well as individuals’ variation across study years. We modeled the effects of primary care models while adjusting for over-dispersion (details in Additional file 2), and physician and patient characteristics on each of the asthma performance indicators. Poisson regression risk ratios were presented with 95% confidence intervals. For comparisons, fee-for-service physicians were used as the reference group because they reflected the original model of health care remuneration used in Ontario. Analyses were performed using SAS version 9.2 [19].

**Table 1:** C**haracteristics of patient population and physician by practice models**

	**Overall**		**Fee-for-service**		**Blended fee-for-service**		**Blended capitation**	
	**Number**	**%**	**Number**	**%**	**Number**	**%**	**Number**	**%**
**2006**								
***Patient characteristics***								
Number of asthma patients	1675282		292222	17.4	1089808	65.1	293252	17.5
Female	890927	53.2	143906	49.2	585260	53.7	161761	55.2
Age groups (years)								
0-4	96542	5.8	33587	11.5	50034	4.6	12921	4.4
5-14	368576	22.0	76955	26.3	232332	21.3	59289	20.2
15-64	1016084	60.7	151812	52.0	680563	62.4	183709	62.6
65+	194080	11.6	29868	10.2	126879	11.6	37333	12.7
Means ± SD	32.8 ± 22.8		28.9 ± 23.3		33.5 ± 22.6		34.2 ± 22.9	
SES by income quintiles								
Quintile 1 - Low	336646	20.1	67381	23.1	213563	19.6	55702	19.0
Quintile 2	335756	20.0	59583	20.4	217530	20.0	58643	20.0
Quintile 3	335221	20.0	55365	18.9	222161	20.4	57695	19.7
Quintile 4	336365	20.1	54480	18.6	220976	20.3	60909	20.8
Quintile 5 - High	325477	19.4	54038	18.5	212220	19.5	59219	20.2
Missing	5817	0.3	1375	0.5	3358	0.3	1084	0.4
Rural residence	174688	10.4	24219	8.3	90771	8.3	59698	20.4
***Physician characteristics***								
Number of physicians	11700		5526	47.2	4603	39.3	1571	13.4
Female	4105	35.1	1816	32.9	1710	37.1	579	36.9
Mean age ± SD	49.6 ± 12.4		50.8 ± 14.4		48.9 ± 10.4		47.8 ± 9.9	
Mean years in practice ± SD	23.7 ± 11.9		24.7 ± 13.2		23.3 ± 10.7		21.9 ± 10.3	
Trained in Canada	8154	69.7	3444	62.3	3371	73.2	1339	85.2
Number of asthma patients on roster								
≤200	2570	22.0	1792	32.4	570	12.4	208	13.2
201-375	2983	25.5	1114	20.2	1352	29.4	517	32.9
≥376	6147	52.5	2620	47.4	2681	58.2	846	53.9
**2010**								
***Patient characteristics***								
Number of asthma patients	1813922		190656	10.5	844836	46.6	778430	42.9
Female	961173	53.0	90427	47.4	443166	52.5	427580	54.9
Age groups (years)								
0-4	77534	4.3	20852	10.9	33098	3.9	23584	3.0
5-14	322417	17.8	48422	25.4	152475	18.0	121520	15.6
15-64	1193641	65.8	102771	53.9	559602	66.2	531268	68.2
65+	220330	12.1	18611	9.8	99661	11.8	102058	13.1
Means ± SD	34.5 ± 22.5		28.6 ± 23.1		34.5 ± 22.3		35.9 ± 22.4	
SES by income quintiles								
Quintile 1 - low	347895	19.2	41894	22.0	169429	20.1	136572	17.5
Quintile 2	356347	19.6	37922	19.9	171875	20.3	146550	18.8
Quintile 3	365799	20.2	36844	19.3	176756	20.9	152199	19.6
Quintile 4	380224	21.0	37315	19.6	175459	20.8	167450	21.5
Quintile 5 - high	357418	19.7	35785	18.8	148943	17.6	172690	22.2
Missing	6239	0.3	896	0.5	2374	0.3	2969	0.4
Rural residence	181703	10.0	12740	6.7	41459	4.9	127504	16.4
***Physician characteristics***								
Number of Physicians	12418		5112	41.2	3487	28.1	3819	30.8
Female	4854	39.1	1886	36.9	1377	39.5	1591	41.7
Mean age ± SD	50.6 ± 12.5		51.2 ± 14.4		51.3 ± 11.3		49.2 ± 10.6	
Mean years in practice ± SD	24.7 ± 12.4		25.1 ± 13.7		25.6 ± 11.6		23.2 ± 11.2	
Trained in Canada	8679	69.9	3305	64.7	2187	62.7	3187	83.5
Number of asthma patients on roster								
≤200	2695	21.7	1516	29.7	576	16.5	603	15.8
201-375	3451	27.8	1043	20.4	963	27.6	1445	37.8
≥376	6272	50.5	2553	49.9	1948	55.9	1771	46.4

### Ethics statement

The study was approved by the institutional review boards at The Hospital for Sick Children, Toronto, Ontario. For the purposes of this research informed consent was not required. The Institute for Clinical Evaluative Sciences (ICES) is named as a prescribed entity in Section 45 of the Personal Health Information Protection Act (PHIPA – Regulation 329/04, Section 18). Under this designation, ICES can receive and use health information without consent for purposes of analysis and compiling statistical information about the Ontario health care system.

## Results

### Study population

Patient and physician characteristics for 2006 and 2010 are shown in Table 1. The majority of patients with asthma were managed by blended fee-for-service physicians. Patient characteristics appear to differ across practice models for sex, age, and rural residence. The fee-for-service model had a smaller percentage of female patients and a larger percentage of younger patients. Detailed data by narrower age groups are provided in Additional file 3. The blended capitation model consisted of more patients living in rural areas. With regard to physician characteristics, the blended capitation model contained more physicians who were trained in Canada. The blended fee-for-service and blended capitation models were more likely to have higher number of asthma patients on their roster than fee-for-service physicians. The proportions of female physicians did not differ across practice models.

### Overall trends of asthma care measured by asthma performance indicators

As seen in Table 2, between 2006 and 2010 there was an overall and gradual increase in the use of spirometry for diagnosis and monitoring of patients with asthma. However, despite this increase in use, in 2010 only half (52.5%) of all asthma diagnoses were confirmed by spirometry, and only 19.1% of asthma patients were monitored annually with spirometry. Overall prescriptions for ICS increased and prescriptions for SABA remained relatively stable over the study period. The percentage of patients being prescribed ≤4 SABA canisters per year increased over time, whereas the percentage of patients being prescribed 13 to 20 and >20 canisters per year decreased over time. Asthma primary care and emergency department visits decreased over time. Primary care and emergency department visits also decreased over time for non-asthma related issues, but not to the same extent as the decline in asthma-specific visits.

**Table 2:** **Overall percent distributions of asthma indicators, 2006 to 2010**

	**2006**		**2007**		**2008**		**2009**		**2010**		**2006 to 2010**		
**Asthma indicators**	**%**	**95% CI**	**%**	**95% CI**	**%**	**95% CI**	**%**	**95% CI**	**%**	**95% CI**	**% change**	**95% CI**	**p-value for trend**
**Process-related indicators**													
Use of spirometry													
To diagnose asthma	46.8	(46.2, 47.5)	48.8	(48.1, 49.5)	49.9	(49.2, 50.7)	51.0	(50.3, 51.8)	52.5	(51.6, 53.5)	12.2	(10.3, 14.0)	<0.001
To monitor asthma	16.4	(16.2, 16.5)	17.0	(16.9, 17.2)	17.2	(17.1, 17.4)	17.7	(17.6, 17.9)	19.1	(18.9, 19.2)	16.5	(15.2, 17.8)	<0.001
**Asthma medication prescriptions (in patients ≥65 years old)**													
Inhaled corticosteroids	72.9	(72.1, 73.7)	74.2	(73.3, 75.0)	74.7	(73.8, 75.5)	75.2	(74.4, 76.1)	76.6	(75.8, 77.5)	5.1	(4.3, 6.0)	<0.001
Short acting β2-agonist	60.5	(59.7, 61.2)	60.7	(59.9, 61.4)	58.2	(57.4, 58.9)	57.8	(57.0, 58.5)	58.5	(57.8, 59.3)	−3.3	(−4.3, −2.1)	<0.001
≤4 canisters per year	56.6	(55.7, 57.6)	57.7	(56.7, 58.6)	60.5	(59.5, 61.5)	61.2	(60.2, 62.3)	61.6	(60.6, 62.7)	8.8	(7.3, 10.5)	<.0001
5-12 canisters per year	30.3	(29.6, 31.0)	30.4	(29.7, 31.1)	29.3	(28.6, 30.0)	29.0	(28.3, 29.7)	29.4	(28.7, 30.1)	−3.0	(−5.5, −0.2)	0.0008
13-20 canisters per year	8.7	(8.3, 9.1)	8.2	(7.9, 8.6)	7.3	(7.0, 7.7)	7.1	(6.8, 7.5)	6.5	(6.2, 6.9)	−25.3	(−29.4, −20.0)	<.0001
>20 canisters per year	4.4	(4.1, 4.6)	3.7	(3.5, 4.0)	2.9	(2.7, 3.1)	2.7	(2.5, 2.9)	2.4	(2.2, 2.6)	−45.5	(−50.5, −39.5)	<.0001
**Outcome-related indicators**													
Asthma specific health services use													
Primary care visits	20.8	(20.8, 20.9)	20.0	(19.9, 20.1)	18.4	(18.3, 18.5)	17.5	(17.4, 17.5)	17.1	(17.0, 17.1)	−17.8	(−18.4, −17.7)	<0.001
Emergency department visits	2.4	(2.4, 2.4)	2.2	(2.2, 2.2)	1.9	(1.9, 1.9)	1.8	(1.8, 1.8)	1.7	(1.7, 1.7)	−29.2	(−30.0, −27.9)	<0.001
Other health services use													
Primary care visits	86.2	(86.1, 86.4)	84.9	(84.8, 85.0)	83.4	(83.3, 83.6)	83.8	(83.7, 83.9)	83.1	(83.0, 83.2)	−3.6	(−3.9, 3.4)	<0.0001
Emergency department visits	26.1	(26.0, 26.1)	25.9	(25.9, 26.0)	25.8	(25.7, 25.8)	25.7	(25.6, 25.8)	25.9	(25.8, 26.0)	−0.8	(−1.1, 0.0)	<0.0001

Note: p-value is based on the Cochran-Armitage test for trend for multiple proportions.

### Crude comparisons of asthma performance indicators by primary care practice models

**Figure 1:**
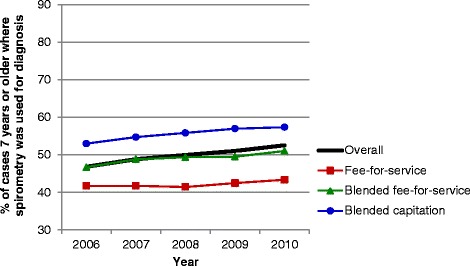
**Spirometry used to establish diagnosis of asthma.** Note: Spirometry was performed on those ≥7 years of age within 3.5 years of the diagnosis date.

**Figure 2:**
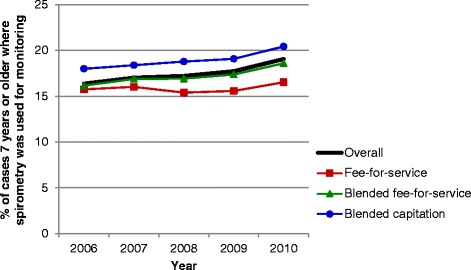
**Spirometry used to monitor asthma.** Note: Spirometry testing was performed on those ≥7 years of age in the past 12 months.

**Figure 3:**
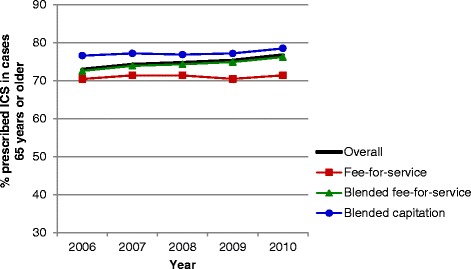
**Inhaled corticosteroid use in the past 12 months for asthma patients >65 years of age.** Note: ICS = inhaled corticosteroids.

**Figure 4:**
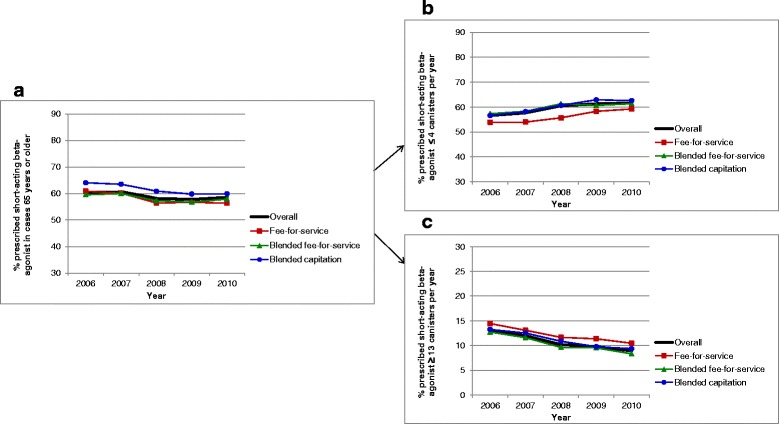
**Short-acting β2-agonist use. a**. Short-acting β2-agonist use in the past 12 months for asthma patients >65 years of age. **b**. Short-acting β2-agonist use ≤4 canisters in the past 12 months for asthma patients >65 years of age. **c**. Short-acting β2-agonist use ≥13 canisters in the past 12 months for asthma patients >65 years of age

**Figure 5:**
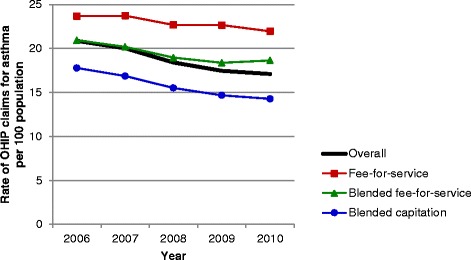
**The rate of primary care visit claims for asthma in the past 12 months.** Note: OHIP = Ontario Health Insurance Plan.

**Figure 6:**
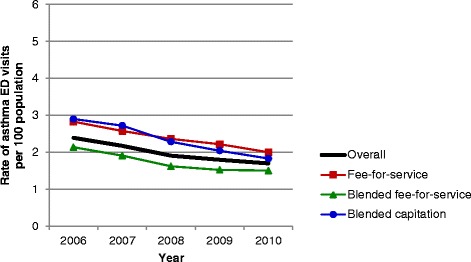
**The rate of emergency department visits for asthma in the past 12 months.** Note: ED = emergency department.

Evaluation of asthma performance indicators demonstrated marked differences over time and among primary care models (Figures 1, 2, 3, 4, 5 and 6). Use of spirometry for asthma diagnosis and monitoring increased over time in all groups; however the increase in the fee-for-service group was the smallest over time for both diagnosis and monitoring (Figures 1 and 2). Use of spirometry for both diagnosis and monitoring was highest over time in the blended capitation group and lowest over time in the fee-for-service group. ICS prescription appears to have increased in the blended-fee-for-service group, while remaining about the same in the other two groups over time; ICS prescription is consistently lower in the fee-for-service group (Figure 3). SABA prescriptions decreased slightly over time in each of the groups (Figure 4a). Prescription of ≤4 SABA canisters per year increased in all groups over time (Figure 4b). Prescription of ≥13 SABA canisters per year decreased in all groups over time (Figure 4c). Primary care and emergency department visits for asthma decreased over time among each of the groups (Figures 5 and 6). Primary care visits were highest in all years for patients in the fee-for-service group, and lowest in the blended capitation group (Figure 5). Emergency department visits were lowest in all years in the blended fee-for-service group (Figure 6).

### Asthma performance adjusted for potential confounders

**Figure 7:**
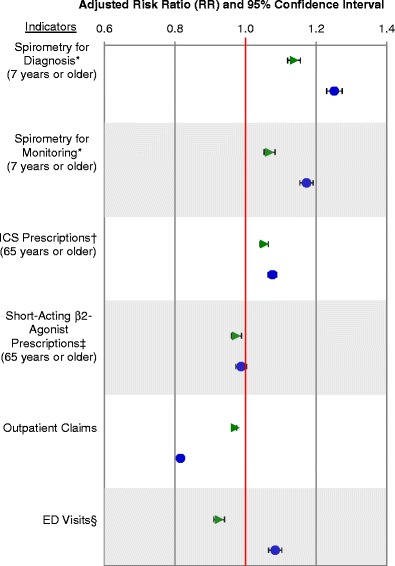
**Adjusted risk ratios of asthma performance indicators comparing primary care practices using fee-for-service practices as a reference.** Note: API=asthma performance indicator. *Performed on those ≥7 years of age, ^†^ICS = inhaled corticosteroids (data only available for persons ≥65 years of age and others eligible for the Ontario Drug Benefits Program), ^‡^Short-acting β2-agonist data only available for persons ≥65 years of age and others eligible for the Ontario Drug Benefits Program, ^§^ED = emergency department. Green triangle = blended fee-for-service practices. Blue circles = blended capitation practices.

As seen in Figure 7, compared to the fee-for-service model, the blended fee-for-service model had higher rates of spirometry use for diagnosis (adjusted risk ratio [RR] = 1.14), spirometry use for monitoring (RR = 1.07), ICS prescriptions (RR = 1.05), and lower rates of SABA prescription (RR = 0.97). In terms of outcome indicators, it had lower rates of emergency department visits (RR = 0.92) and slightly lower rates of outpatient claims (RR = 0.97).

Compared to fee-for-service models, the blended capitation model also had higher rates of spirometry use for diagnosis (RR = 1.25), spirometry for monitoring (RR = 1.17), and ICS prescriptions (RR = 1.08). Their rate of SABA prescription did not differ from the fee-for-service model. In terms of outcome indicators, their rate of outpatient claims (RR = 0.82) are much lower than both the fee-for-service and blended fee-for-service models, but their patients’ rates of emergency department visits (RR = 1.08) were higher than both the fee-for-service and blended fee-for-service models.

## Discussion

Overall, quality of asthma care, as represented by performance indicators, has improved over time. In general, patient enrolment models (i.e., blended fee-for-service and blended capitation models) appear to provide higher quality of asthma care compared to traditional fee-for-service models based on the analysis of asthma performance indicators. The blended-fee-for service group stood out from both the fee-for-service and blended capitation in terms of outcome indicators, namely by having a lower rate of emergency department visits.

While the blended capitation model had higher rates of use of spirometry for diagnosis and monitoring, and higher rates of ICS prescriptions, they had lower rates of outpatient visits and higher rates of emergency department visits. These findings may be related to the fact that the blended capitation model had more patients living in rural areas. However, these results stood out even after adjusting for patient characteristics. Thus, we can suggest other factors that may have played a role, such as co-morbidities, poor compliance to medication, limited access to primary care because of geographic location or office hours, poor availability of after-hours medical assistance, patient education, or limitations of the process indicators to measure all variables involved with quality of care.

Recent studies in Ontario have also shown quality of care to be affected by primary care delivery models. Glazier et al. also observed that patients followed by physicians paid by the blended capitation model had higher than expected emergency department visits than fee-for-service physicians, whereas the patients in the salaried model were sicker but had substantially lower emergency department visit rates than expected [20]. The study by Glazier et al. showed that different primary care models served different patient populations, with non-salaried health centre physicians caring for more advantaged populations, and salaried physicians caring for more disadvantaged populations. These data suggest that pay structure may not be the only reason for differences in quality of care – individual patient characteristics not measured with administrative data, specific geographical location and practice infrastructure may partially explain the observed differences. Further, the use of performance indicators may be influenced by financial incentives within the different models.

These findings are relevant on an international level, where there is a trend towards payment tied to performance and bundled primary care systems. In the United Kingdom, primary care has moved towards group-based practices with pay-for-performance compensation, in which performance is measured using a number of quality indicators [21]. In the United States, where compensation is predominantly fee-for-service, policy recommendations have suggested a shift towards more bundled payment systems, in which payment is also tied to performance [22].

Despite an overall increase in quality of asthma care over time, there remains much room for improvement. National guidelines recommend that all asthma cases should be diagnosed using spirometry (in individuals old enough to reliably perform spirometry); however, our findings show that only half of patients with asthma had their asthma diagnosed using spirometry. Furthermore, guidelines describe the necessity of regular monitoring of asthma using spirometry, yet less than one-fifth of patients in our study underwent spirometry in the past year.

A few limitations of this study should be noted. The health administrative data definition of asthma may be subject to potential misclassification when compared to clinical evaluation by a physician, compounded by the fact that physicians do not always accurately diagnose asthma. When physicians submit claims, they enter an asthma diagnostic code. This is often based on patient symptoms, such as wheeze and cough, and can be subject to error. Further, as a large number of individuals were older than 65 years of age, it is possible that they had COPD in addition to asthma, or that the symptoms they were presenting with were those of COPD, thus there is the potential for misclassification or type 2 error. While some studies have shown asthma to be overdiagnosed by physicians, others have shown it to be underdiagnosed; it is therefore not clear how such possible misdiagnosis influenced our results [23-25]. Other research comparing asthma health administrative and survey data and found that, while there were differences in absolute values, health administrative data were consistent over time and therefore reliable for studying trends [26].

As suggested by Devlin and Sarma [27], physicians may self-select into different primary care models based on personal preferences and unobserved characteristics leading to potential selection bias. We included physician characteristics in our regression analysis to help reduce, though not eliminate, selection bias. In future, formal approaches such as propensity score matching used by Kantarevic et al. [28] or the difference-in-difference matching strategy employed by Kantarevic et al. [29] and Li et al. [30] should be considered to control for potential selection bias through the inclusion of physician individual fixed effects.

We were unable to include indicators for the entire population for analysis (e.g., prescription drug data was only available for individuals 65 years or older). In addition, data were available on dispensing practices but not on whether participants adhered to taking the medication. Further, use of portable peak expiratory flow meters as an objective measure of lung function to diagnose and monitor asthma was not captured by administrative billing data.

While this is not a randomized control trial, and we cannot guarantee that patient and physician groups were similar, we did adjust for many patient and physician characteristics in our analyses. Further to the discussion of differences between models, it cannot be determined with certainty whether or not more frequent use of indicators resulted in better patient outcomes.

## Conclusions

Quality of asthma care has improved over time. Patient enrolment models appear to provide higher quality of asthma care processes compared to traditional fee-for-service model. Future improvements in quality of asthma care should focus on increasing the use of spirometry for asthma diagnosis and monitoring. Future research should continue to use administrative data to track asthma indicators on a population level to provide a foundation for continuous quality improvement in the provision of asthma care, as well as examine the direct relationship between use of asthma indicators and patient outcomes.

## Additional files

Additional file 1:
**Ontario Asthma Surveillance Information System Administrative Databases.**
Additional file 2:
**Method Details.**
Additional file 3:
**Detailed age distribution of patient population by practice models.**

## Abbreviations

COPD: Chronic obstructive pulmonary disease
GEE: Generalized estimating equations
ICD: International Classification of Diseases
ICES: Institute for Clinical Evaluative Sciences
ICS: Inhaled corticosteroids
MOHLTC: The Ontario Ministry of Health and Long-Term Care
SABA: Short-acting β_2_-agonists
RR: Adjusted risk ratio

## References

[1] Russell GM, Dahrouge S, Hogg W, Geneau R, Muldoon L, Tuna M (2009). Managing chronic disease in ontario primary care: the impact of organizational factors. Ann Fam Med.

[2] Family Practice Compensation Models. [http://www.healthforceontario.ca/en/Home/Physicians/Training_%7C_Practising_in_Ontario/Physician_Roles/Family_Practice_Models/Family_Practice_Compensation_Models]

[3] To T, Cicutto L, Degani N, McLimont S, Beyene J (2008). Can a community evidence-based asthma care program improve clinical outcomes? A longitudinal study. Med Care.

[4] To T, McLimont S, Wang C, Cicutto L (2009). How much do health care providers value a community-based asthma care program? – a survey to collect their opinions on the utilities of and barriers to its uptake. BMC Health Serv Res.

[5] To T, Daly C, Feldman R, McLimont S (2012). Results from a community-based program evaluating the effect of changing smoking status on asthma symptom control. BMC Public Health.

[6] To T, Dell S, Dick PT, Cicutto L, Harris JK, MacLusky IB (2006). Case verification of children with asthma in Ontario. Pediatr Allergy Immunol.

[7] Gershon AS, Wang C, Guan J, Vasilevska-Ristovska J, Cicutto L, To T (2009). Identifying patients with physician diagnosed asthma in health administrative databases. Can Respir J.

[8] To T, Guttmann A, Lougheed MD, Gershon AS, Dell SD, Stanbrook MB (2010). Evidence-based performance indicators of primary care for asthma: a modified RAND Appropriateness Method. Int J Qual Health Care.

[9] Gershon AS, Victor JC, Guan J, Aaron SD, To T (2012). Pulmonary function testing in the diagnosis of asthma: a population study. Chest.

[10] Lougheed M, Lemière C, Dell S, Ducharme F, Fitzgerald J, Leigh R (2010). Canadian Thoracic Society Asthma Management Continuum–2010 Consensus Summary for children six years of age and over, and adults. Can Respir J.

[11] Lougheed MD, Lemiere C, Ducharme FM, Licskai C, Dell SD, Rowe BH (2012). Canadian Thoracic Society 2012 guideline update: Diagnosis and management of asthma in preschoolers, children and adults. Can Respir J.

[12] Lynd L, Sandford A, Kelly E, Paré P, Bai T, Fitzgerald J (2004). Reconcilable differences: a cross-sectional study of the relationship between socioeconomic status and the magnitude of short-acting beta-agonist use in asthma. Chest.

[13] Lynd L, Guh D, Paré P, Anis A (2002). Patterns of inhaled asthma medication use: a 3-year longitudinal analysis of prescription claims data from British Columbia, Canada. Chest.

[14] Glazier R, Zagorski B, Rayner J (2012). Comparison of Primary Care Models in Ontario by Demographics, Case Mix and Emergency Department Use, 2008/09 to 2009/10. ICES Investigative Report.

[15] Matheson FI, Moineddin R, Dunn JR, Creatore MI, Gozdyra P, Glazier RH (2006). Urban neighborhoods, chronic stress, gender and depression. Soc Sci Med.

[16] Gershon A, Guan J, Wang C, Victor J, To T (2012). Describing and quantifying asthma comorbidity [corrected]: a population study. PLoS ONE.

[17] Gershon A, Wang C, Guan J, Vasilevska-Ristovska J, Cicutto L, To T (2009). Identifying individuals with physician diagnosed COPD in health administrative databases. COPD.

[18] Jennison C, Turnbull B (1993). Sequential equivalence testing and repeated confidence intervals, with applications to normal and binary responses. Biometrics.

[19] SAS Institute Inc (2006). SAS/STAT User's Guide, Version 9.

[20] Glazier RH, Zagorski BM, Rayner J (2012). Comparison of Primary Care Models in Ontario by Demographics, Case Mix and Emergency Department Use, 2008/09 to 2009/10.

[21] Roland M (2004). Linking physicians' pay to the quality of care - a major experiment in the United Kingdom. N Engl J Med.

[22] Shih A, Davis K, Schoenbaum S, Gauthier A, Nuzum R, McCarthy D (2008). Organizing the U.S. Health Care Delivery System for High Performance. The Commonwealth Fund.

[23] Aaron SD, Vandemheen KL, Boulet LP, McIvor RA, Fitzgerald JM, Hernandez P (2008). Overdiagnosis of asthma in obese and nonobese adults. CMAJ.

[24] Stupka E, de Shazo R (2009). Asthma in seniors: Part 1. Evidence for underdiagnosis, undertreatment, and increasing morbidity and mortality. Am J Med.

[25] Quinn K, Shalowitz M, Berry C, Mijanovich T, Wolf R (2006). Racial and ethnic disparities in diagnosed and possible undiagnosed asthma among public-school children in Chicago. Am J Public Health.

[26] Huzel L, Roos LL, Anthonisen NR, Manfreda J (2002). Diagnosis asthma: The fit between survey and administrative database. Can Respir J.

[27] Devlin RA, Sarma S (2008). Do physicians remuneration schemes matter? The case of Canadian family physicians. J Health Econ.

[28] Kantarevic J, Kralj B, Weinkauf D (2011). Enhanced fee-for-service model and physician productivity: evidence from Family Health Groups in Ontario. J Health Econ.

[29] Kantarevic J, Kralj B (2013). Link between pay for performance incentives and physician payment mechanisms: evidence from the diabetes management incentive in ontario. Health Econ.

[30] Li J, Hurley J, Decicca P, Buckley G. Physician response to pay-for-performance: evidence from a natural experiment. Health Econ. 2013. 10.1002/hec.2971 23861240

