# Supplementary material for: Quality of asthma care under different primary care models in Canada: a population-based study
Source: BMC Fam Pract. 2015 Feb 14;16:19. doi: 10.1186/s12875-015-0232-y (PMC4336688; doi:10.1186/s12875-015-0232-y)
Supplement: Additional file 1: — Ontario Asthma Surveillance Information System Administrative Databases. [file 12875_2015_232_MOESM1_ESM.pdf]

Additional file 1: Ontario Asthma Surveillance Information System Administrative Databases

1. Ontario Health Insurance Plan Database: contains information on fee-for-service billings for physician services rendered;
2. National Ambulatory Care Reporting System: contains data for emergency department and outpatient visits;
3. Canadian Institute for Health Information Discharge Abstract Database: records primary diagnosis and up to 24 secondary diagnoses for all hospitalizations;
4. Ontario Registered Persons Database: includes information on sex, birth date, residence postal code, and, if applicable, date of death.
