# Supplementary material for: Quality of asthma care under different primary care models in Canada: a population-based study
Source: BMC Fam Pract. 2015 Feb 14;16:19. doi: 10.1186/s12875-015-0232-y (PMC4336688; doi:10.1186/s12875-015-0232-y)
Supplement: Additional file 2: — Method Details. [file 12875_2015_232_MOESM2_ESM.pdf]

Additional file 2: Method Details

Poisson regression analysis: Since our outcomes of interest were counts, the Poisson regression model was the method of choice. An "off-set" parameter was included in the regression to model the count as a rate by dividing the counts over population denominator. The goodness-of-fit of the Poisson model and the indication of over-dispersion was measured using the value divided by degrees of freedom (VF). All the VFs in our regression models were close to 1 indicating the absence of over-dispersion. In our multivariable analysis, we used the two-level Poisson regression with GEE (Generalized Estimating Equation) to account for both the clustering of patients within primary care models as well as adjusting for individual patient's variation across study years. Multicollinearity was examined using the variance inflation factor (VIF) and none was higher than 5 suggesting no multicollinearity in variables included in the final regression model selected.
