# Supplementary material for: Quality of asthma care under different primary care models in Canada: a population-based study
Source: BMC Fam Pract. 2015 Feb 14;16:19. doi: 10.1186/s12875-015-0232-y (PMC4336688; doi:10.1186/s12875-015-0232-y)
Supplement: Additional file 3: — Detailed age distribution of patient population by practice models. [file 12875_2015_232_MOESM3_ESM.docx]

Additional file 3: Detailed age distribution of patient population by practice models

|  | Overall | | Fee-for-service | | Blended fee-for-service | | Blended Capitation | |
| --- | --- | --- | --- | --- | --- | --- | --- | --- |
|  | Number | % | Number | % | Number | % | Number | % |
| **2006** |  |  |  |  |  |  |  |  |
| ***Patient Characteristics*** |  |  |  |  |  |  |  |  |
| Number of Asthma Patients | 1675282 |  | 292222 | 17.4 | 1089808 | 65.1 | 293252 | 17.5 |
| 0-4 | 96542 | 5.8 | 33587 | 11.5 | 50034 | 4.6 | 12921 | 4.4 |
| 5-9 | 162734 | 9.7 | 37319 | 12.8 | 100772 | 9.2 | 24643 | 8.4 |
| 10-14 | 205842 | 12.3 | 39636 | 13.6 | 131560 | 12.1 | 34646 | 11.8 |
| 15-19 | 183393 | 10.9 | 29560 | 10.1 | 120463 | 11.1 | 33370 | 11.4 |
| 20-34 | 308799 | 18.4 | 45769 | 15.7 | 206732 | 19.0 | 56298 | 19.2 |
| 35-49 | 297960 | 17.8 | 42498 | 14.5 | 202875 | 18.6 | 52587 | 17.9 |
| 50-64 | 225932 | 13.5 | 33985 | 11.6 | 150493 | 13.8 | 41454 | 14.1 |
| 65+ | 194080 | 11.6 | 29868 | 10.2 | 126879 | 11.6 | 37333 | 12.7 |
| **2010** |  |  |  |  |  |  |  |  |
| ***Patient Characteristics*** |  |  |  |  |  |  |  |  |
| Number of Asthma Patients | 1813922 |  | 190656 | 10.5 | 844836 | 46.6 | 778430 | 42.9 |
| Age Groups (years) |  |  |  |  |  |  |  |  |
| 0-4 | 77534 | 4.3 | 20852 | 10.9 | 33098 | 3.9 | 23584 | 3.0 |
| 5-9 | 140181 | 7.7 | 25520 | 13.4 | 65275 | 7.7 | 49386 | 6.3 |
| 10-14 | 182236 | 10.0 | 22902 | 12.0 | 87200 | 10.3 | 72134 | 9.3 |
| 15-19 | 215525 | 11.9 | 21686 | 11.4 | 98944 | 11.7 | 94895 | 12.2 |
| 20-34 | 391130 | 21.6 | 34685 | 18.2 | 182714 | 21.6 | 173731 | 22.3 |
| 35-49 | 312178 | 17.2 | 24020 | 12.6 | 149262 | 17.7 | 138896 | 17.8 |
| 50-64 | 274808 | 15.1 | 22380 | 11.7 | 128682 | 15.2 | 123746 | 15.9 |
| 65+ | 220330 | 12.1 | 18611 | 9.8 | 99661 | 11.8 | 102058 | 13.1 |
